# Training demands on clerk burnout: determining whether achievement goal motivation orientations matter

**DOI:** 10.1186/s12909-016-0742-x

**Published:** 2016-08-22

**Authors:** Chia-Der Lin, Blossom Yen-Ju Lin

**Affiliations:** 1 Department of Education, Department of Otolaryngology, China Medical University Hospital, Taichung, Taiwan, Republic of China; 2 School of Medicine, Medical Sociology, China Medical University, No.91, Hsueh-Shih Road, Taichung, 40402 Taiwan, Republic of China

**Keywords:** Achievement goal motivation, Clerkship, Training demands, Burnout, Medical students

## Abstract

**Background:**

In the education field, learning experiences are considered learners’ properties and are viewed as a key determinant in explaining learners’ learning processes, especially for training novices such as clerks with varying levels of commitment to the medical profession. This study explored whether clerks’ achievement goal motivation orientations might buffer the negative well-being to a certain extent, considering their training demands during clinical training.

**Methods:**

Ninety-four clerks at a tertiary medical center were longitudinally traced during their 2-year clerkship spanning from September 2013 to April 2015. Web-based, validated, structured, self-administered questionnaires were used to evaluate the clerks’ properties of achievement goal motivation orientation and personal background at the beginning of the clerkship. Regular surveys were conducted to evaluate their perceptions of training demands and burnout at each specialty rotation. Overall, 2230 responses were analyzed, and linear mixed-effects models were used to examine the repeated measures of the clerks.

**Results:**

The results revealed that higher perceived psychological and physical demands of training were related to higher perceived burnout during the 2-year clerkship. Although both the clerks’ task and ego orientations were related to reduced burnout (direct effects), only task orientation was indicated to exert a buffering effect on their perception of physical demands on burnout in the 1st year of the clerkship.

**Conclusions:**

Considering the negative effects of training demands (psychological and physical), we observed a limited effect of the task achievement motivation orientation of medical students; therefore, additional studies might focus on strategies to facilitate medical students in clerkships in addressing both the psychological and physical demands inherent in training workplaces to improve their learning experience and well-being.

## Background

From the perspective of medical education, the stage of clinical training is a crucial transition period for medical students because it represents the time when their role substantially changes from that of students to that of doctors who must bear considerable social responsibility and demand. This is also the time when clerks undergo vigorous challenges on their intelligence, physical strength, and stamina [1–3]. A challenge for clerks is that although they are assigned the role and responsibility of doctors in clinical workplaces in which patient care is highly emphasized, they tend to consider themselves as learners [4]. However, the real work environment of medical institutions can impose extreme stress that adversely affects the well-being of medical students during clinical training [5]. Challenges include adapting to heavy workloads [6–9], which has been proven to detract from the training of medical students [10]. A state of negative well-being may negatively influence the skills learned by medical students and the quality of medical care, which may consequently engender medical disputes.

By applying the Karasek and Theorell job demand-control-support model as a framework [11], several studies have revealed that high work demands, such as high workloads, demonstrated the most pronounced relationship with somatic complaints for resident physicians [12], with radiologists’ metabolic syndromes [13], and with junior doctors’ intentions to leave clinical practice [14]. In addition, job demands that derived job strain were determined to be related to physician burnout [15] and poor quality of health care such as self-assessment performance, service quality, and error frequency [16]. However, previous studies on clerkship education have focused more on formal and standardized elements of the taught and assessed curriculum, affective supports from medical and nonmedical professional interactions, and pedagogic elements, such as teaching, supervision, and precepting [17], rather than on how clerks might perceive their work demanded from training in the workplaces.

In the education field, learning experiences are regarded as learners’ properties and are considered a key determinant in explaining learners’ learning processes, particularly for training novices such as clerks with varying levels of commitment to the medical profession [18]. Achievement goal motivation theory is conventionally used to discuss a learner’s needs for motivations of achievements and is currently primarily adopted to explain the correlation between learning and task performance [19]. Achievement goal motivation theory has been explained disparately by 2 schools of scholars. One school regards goals as task oriented (mastery goals or learning goals) [20–22], implying that learners are concerned about how to increase their knowledge, skills, and competence. In other words, learners focus on the intrinsic value of learning, which is their proficiency on and understanding of the learning content [20, 23]. Such types of learners believe that outcomes are strongly related to efforts; therefore, they must invest a considerable amount of effort to achieve success [24]. The other school of scholars regards goals as ego orientation (performance goals) [20, 22], which center on learner abilities. Such types of learners believe that outcomes are strongly related to abilities; therefore, they set goals from the perspective that their abilities are superior to those of others [20]. Research reveals that achievement goal motivation theory might be a powerful framework for understanding student engagement, persistence on tasks, and academic reliance [25]. Gardner et al. examined 3rd-year medical students who were in their clerkship and revealed that the clerk goal orientation must be considered during the curriculum development to maximize surgical education values [18]. Madjar et al. also revealed that among 1st-year medical students in a doctor–patient communication course, task orientation was positively related to perceived psychosocial abilities and negatively related to low frustration tolerance, whereas ego orientation was related to low frustration tolerance [26].

We might argue that although training demands might undermine the well-being of medical students to an extent during clinical training, the properties of medical students might partly buffer their perceptions about the training experience. If the medical student personal properties (ie, achievement goal motivation orientations) could be counted, mentors and clinical teachers must understand their trainees’ properties and provide necessary assistance and mentoring during clinical training. Therefore, this study proposed 2 research hypotheses: (1) Higher training demands for clerks in clinical workplaces are associated with poor well-being in those clerks, and (2) clerks’ achievement goal motivation orientations might buffer their negative well-being to an extent, considering their training demands in clinical workplaces.

## Methods

This was a prospective cohort web-based questionnaire study.

### Participants and data collection

Our study comprised one cohort of medical students in their 5th and 6th years as clerks in a 7-year medical education program at China Medical University, Taiwan between September 2013 and April 2015. Medical students in their clerkships in Taiwan are equivalent to 3rd- or 4th-year medical students undergoing clinical training in the 4-year graduate MD programs in Western countries. Of 190 clerks who attended recruitment meetings, 111 (58.4 %) agreed to participate in the study and provided written informed consent. The participants first completed a validated, structured, self-administered questionnaire describing their properties of achievement goal motivation orientation and personal background at the beginning of the clerkship in September 2013. Subsequently, until April 2015, they regularly answered web-based validated, structured, self-administered questionnaires that evaluated their perceptions of training demands and burnout at each specialty rotation. All the medical students in the clerkship had the same arrangement of clinical curricula (rotated clinical specialties), but they might be in different rotation orders. The clinical specialties covered in the 1st-year clerkship were internal medicine (cardiology, chest medicine, haematology & oncology, infectious diseases, nephrology, gastroenterology, general medicine, and metabolism & endocrinology/rheumatology), psychiatry, neurology, surgery (general surgery, chest surgery, cardiovascular surgery, colon & rectal surgery, paediatric surgery, and trauma), obstetrics and gynaecology, paediatrics, and radiology; those covered in the 2nd-year clerkship were anaesthesiology, orthopaedics, neurosurgery, plastic surgery, urology, dermatology, rehabilitation medicine, ophthalmology, otolaryngology, family medicine, emergency medicine, and nuclear medicine/pathology/laboratory medicine/radiation oncology.

All participants were free to decide whether to complete the survey each time, and each participant presented various responses during the study period. Participants with at least 10 responses to our routine study survey, indicating their longitudinal participation for at least 6 months, were included in this study. Overall, 94 clerks who provided a total of 2230 responses (1357 and 873 responses during the 1st and 2nd clerkship years, respectively) were included in our analysis [27].

### Study development and administration

#### Training demand of clerks’ individual specialty rotations

Training demands across individual specialty rotations as perceived by the clerks were measured through 10 question items rated on the scales 1 (*strongly disagree*), 2 (*disagree*), 3 (*fair*), 4 (*agree*), and 5 (*strongly agree*); these items were adapted from the Job Content Questionnaire (JCQ), a tool for psychosocial job assessment, implemented by Karasek et al. [28]. Two constructs, namely psychological and physical demands, were examined in this study. Psychological demands of work refer to task control and skill use (decision latitude) related to mental workloads, organizational constraints on task completion, and conflicting demands, whereas physical demands involve not only mental but also physical loads [29]. Factor analyses revealed psychological (6 items) and physical (4 items) demands with factor loadings of 0.70–0.84 and 0.81–0.91, respectively. The Cronbach alpha values were 0.90 and 0.94 for the psychological and physical demands, respectively (Table 1). The factor scores of the psychological and physical training demands were calculated using a regression model and were used for further analyses.

**Table 1:** Clerks’ achievement goal motivation orientations, perceived rotated specialty training demands, and burnout in clinical workplaces

Variable	Mean	SD	Factor loading	Cronbach’s alpha
Clerk achievement goal motivation orientation (score: 1–5)				
*Task orientation:* I feel most successful	4.42	0.49		0.88
1. Reaching a goal	4.43	0.58	0.77	
2. Showing personal improvement	4.44	0.54	0.86	
3. Performing to the best of ability	4.39	0.64	0.76	
4. Working hard	4.28	0.74	0.68	
5. Overcoming difficulties	4.50	0.60	0.77	
6. Mastering something I could not do before	4.45	0.58	0.78	
*Ego orientation:* I feel most successful	3.93	0.79		0.91
7. I am the best	4.19	0.88	0.85	
8. I do better than opponents	4.15	0.87	0.84	
9. I show others I am the best	3.86	1.06	0.81	
10. I am clearly superior	3.74	1.02	0.81	
11. I beat other people	3.43	1.01	0.78	
12. I accomplish something others cannot do	4.19	0.78	0.77	
Clerk perceived rotated specialty training demands (score: 1–5)				
*Psychological demands*	2.56	0.82		0.90
1. Fast-paced work environment	2.65	1.04	0.84	
2. Hard work	2.47	0.95	0.84	
3. Busy at work	2.52	0.98	0.77	
4. Intense work concentration	3.01	1.00	0.75	
5. Not enough sleeping time	2.35	1.01	0.70	
6. Work fatigue	2.37	1.03	0.70	
*Physical demands*	2.00	0.79		0.94
7. Awkward body position	1.99	0.86	0.91	
8. Awkward arm position	1.97	0.84	0.91	
9. Lifting heavy load	1.94	0.82	0.88	
10. Rapid physical activity	2.09	0.91	0.81	
Clerk burnout in the rotated specialties^a^ (score: 10–50)				
First-year clerkship	24.35	5.11		
Second-year clerkship	23.55	5.57		

^a^Significant difference (*P* < .05) was observed between the first- and second-year clerkship students examined using mixed modelling that accounted for repeated measures of individual participant burnout

#### Clerk achievement goal motivation orientation

The clerks’ levels of achievement goal motivation orientation were measured using the Perception of Success Questionnaire containing 12 items [30, 31] that are rated on 1 (*strongly disagree*), 2 (*disagree*), 3 (*fair*), 4 (*agree*), and 5 (*strongly agree*)*.* Factor analysis indicated two constructs, namely task orientation (6 items) and ego orientation (6 items), with factor loadings of 0.68–0.86 and 0.77–0.85, respectively. The Cronbach alpha values were 0.88 and 0.91 for task and ego orientations, respectively (Table 1). The factor scores of task and ego orientations were calculated by the regression model and were used for additional analyses.

#### Clerk burnout for each specialty rotation

The clerk burnout was measured using the Professional Quality of Life: Compassion Satisfaction and Fatigue Version 5 scale [32], which has been gradually adopted in medical studies [27, 33–35]. Burnout is a negative emotion associated with feelings of hopelessness and difficulty in managing work or performing a job effectively, and it was measured using 10 items answered on a 5-point scale for our study [35]. The 10 items enquired how frequently the participants experienced various emotions, and the responses were 1 (*never*), 2 (*seldom*), 3 (*sometimes*), 4 (*often*), and 5 (*always*). The Cronbach alpha value was 0.77. The burnout score calculation comprises the following steps: (1) reversing the necessary items for burnout, and (2) summing the items for burnout [36]. The summed score was used for further analysis. Participant burnout was regularly measured after each clinical specialty rotation during clerkship training from September 2013 to April 2015 [27].

#### Clerk characteristics

In this study, clerk gender and age were included as confounding factors, which were determined with regard to achievement goal motivation orientations [37, 38].

### Statistical analysis

Descriptive analyses were performed to examine the clerks’ perceived achievement goal motivation orientations (ie, ego and task) at the baseline and burnout at various clinical specialty rotations. Because each participant (clerk) was not an independent cohort (ie, their responses were treated as correlated data), did not exhibit unequal variance, and involved an unequal number of repetitions, linear mixed-effects models were employed during both clerkship training years [39, 40]. The clerks’ repeated burnout was considered as a dependent variable, training demands in each specialty rotation as independent variables, achievement goal motivation orientations as moderators—2 × 2 interaction terms of 2 types of achievement goal motivation orientations (ego and task) and 2 types of training demands (physical and psychological)—and demographic characteristics (age and gender) as covariates. All analyses were performed using SPSS (Version 20.0).

## Results

We included 94 participants (49 men [52 %], 45 women [48 %]; average age: 23 y) in our study. In terms of achievement goal motivation orientations (Table 1), the clerks exhibited a mean score of 4.42 for the 6 items of task orientation and 3.93 for the 6 items of ego orientation, with a score of 3 indicating a neutral value.

We obtained a total of 2230 responses from the 94 clerks; through the 2-year clerkship training on average, the score of the clerks’ perceived psychological demands for the individual rotated specialties was 2.56 and that for physical demands was 2.00, with a score of 3 representing a neural value (Table 1). When the period was further broken down into 2 individual years of clerkship training, the results revealed that the clerks’ perceptions of psychological demands in the 1st year of clerkship training were slightly higher than those in the 2nd year of the clerkship (*P* < .05), but not physical demands through the 2-year clerkship (*P* > .05).

Regarding the clerks’ perceived burnout for the individual rotated specialties on average, the results revealed clerk burnout scores of 24.35 and 23.55 in the 1st and 2nd years of the clerkship, respectively, and the perceived burnout in the 1st year of the clerkship was slightly higher than that in the 2nd year of the clerkship (*P* < .05) (Table 1).

### Hypotheses 1 testing: Higher training demands for clerks in clinical workplaces are associated with poor well-being in those clerks

In the 1st and 2nd years of the clerkship (Table 2), considering age and gender as covariates, the linear mixed-effects models revealed that higher perceived psychological and physical demands were related to higher burnout (*P* < .05). Being a man and younger were related to increased burnout (*P* < .05).

**Table 2:** Relationships of training demands over specialty rotations on clerk 1st-year and 2nd-year burnout in clinical workplaces

Parameters	Estimates	SE	*P* value	95 % confidence interval	
				Lower	Upper
*First-year clerkship*					
Gender (reference: female)	0.67	0.26	0.01	0.16	1.18
Age	-0.29	0.05	0.00	-0.39	-0.19
Psychological demand (PSY)	0.81	0.13	0.00	0.56	1.06
Physical demand (PHY)	1.69	0.12	0.00	1.45	1.93
*Second-year clerkship*					
Gender (reference: female)	1.06	0.35	0.00	0.36	1.75
Age	-0.43	0.07	0.00	-0.58	-0.29
Psychological demand (PSY)	0.68	0.18	0.00	0.32	1.04
Physical demand (PHY)	1.52	0.18	0.00	1.17	1.87

### Hypotheses 2 testing: The clerk achievement goal motivation orientation might buffer their negative well-being to a certain extent, considering their training demands in clinical workplaces

To examine the buffering effect of the clerks’ achievement goal motivation orientation in the 1st and 2nd years of the clerkship (Table 3), considering age and gender as covariates, the linear mixed-effect models revealed that higher perceived psychological and physical demands were related to higher perceived burnout (*P* < .05), whereas the clerks’ task achievement goal motivation orientation and ego achievement goal motivation orientation were both related to reduced burnout (*P* < .05, direct effect). However, only the clerks’ task achievement goal orientations exerted a buffering effect on the relationship between perceived physical demands and burnout across rotated specialties in the 1st-year clerkship (*P* < .05).

**Table 3:** Buffering (moderating) effect of the clerks’ achievement goal motivation orientations on training demands and burnout in clinical workplaces

Parameters	Estimates	SE	*P* value	95 % confidence interval	
				Lower	Upper
*First-year clerkship*					
Gender (reference: female)	0.49	0.26	0.06	-0.02	1.01
Age	-0.27	0.05	0.00	-0.38	-0.17
Psychological demand (PSY)	0.78	0.13	0.00	0.53	1.04
Physical demand (PHY)	1.51	0.13	0.00	1.25	1.76
Task orientation (TASK)	-0.40	0.13	0.00	-0.66	-0.14
Ego orientation (EGO)	-0.40	0.13	0.00	-0.66	-0.14
PSY*TASK	0.06	0.14	0.68	-0.22	0.34
PSY*EGO	0.08	0.13	0.53	-0.17	0.34
PHY*TASK	-0.28	0.13	0.03	-0.53	-0.02
PHY*EGO	-0.18	0.12	0.13	-0.41	0.05
*Second-year clerkship*					
Gender (reference: female)	0.89	0.36	0.01	0.19	1.58
Age	-0.42	0.07	0.00	-0.57	-0.28
Psychological demand (PSY)	0.65	0.19	0.00	0.28	1.01
Physical demand (PHY)	1.27	0.19	0.00	0.90	1.64
Task orientation (TASK)	-0.63	0.20	0.00	-1.02	-0.24
Ego orientation (EGO)	-0.85	0.21	0.00	-1.27	-0.44
PSY*TASK	-0.17	0.21	0.42	-0.59	0.25
PSY*EGO	0.19	0.20	0.36	-0.21	0.59
PHY*TASK	0.20	0.19	0.30	-0.18	0.58
PHY*EGO	0.12	0.19	0.54	-0.26	0.49

## Discussion

A total of 2230 responses from 94 clerks over a 2-year clerkship period were analyzed to determine (1) the relationship between the training demands (psychological and physical demands) and the clerk burnout and (2) to identify whether the clerks’ achievement goal motivation orientation might exert a buffering effect on the relationship between perceived training demands and burnout. As expected, higher perceived psychological and physical demands were related to the clerk higher perceived burnout across the 2-year clerkship. Although both the clerks’ task and ego orientations were related to reduced burnout (direct effects), only task orientation was indicated to have a buffering effect on the clerks’ perception of physical demands on their burnout in the 1st-year clerkship training.

In our study, the physical and psychological demands perceived by the clerks were related to their increased burnout (*P* < .05) in the 1st and 2nd years of the clerkship. Our results verify the argument of the job demand-control model that adverse working conditions associated with physical and psychological demands are potential risk factors for workplace trainees, and similar findings have been reported by studies of resident physician well-being [12] and junior doctor leaving intentions [14].

We further examined the items of physical and psychological demands individually, as presented in Table 1, with a score of 3 representing a neutral value. We observed that the scores of all the training demand items were lower than the neural value, except for that of the item measured as “Intense work concentration (Item 4)”; nevertheless, the scores of the 6 items of psychological demands (mean 2.35–3.01) were higher than those of the 4 items of physical demands (mean 1.94–2.09). Studies have argued that the pace and intensity of the clerkship experience were new for medical students in clerkship, resulting in such students feeling mentally and physically exhausted [41, 42]. Moreover, Wenrich et al. indicated that medical students had higher expectations of preparing themselves for gaining skills required in clinical practice, compared with clerkship faculty, in terms of basic clinical cognitive and technical skills such as communication skills, history taking, system review completion, full physical exam execution, oral case presentation, and write-up completion [43]. We might suggest that hospitals conduct an orientation at the beginning of clerkships for medical students who initiated their clinical training about what to expect to reduce their first-contact shock. Such an orientation could include information about the clarification of skill expectations that might alleviate student anxiety about their clerkships and enhance their learning [6]. Moreover, the needs of effective time management training for ensuring reduced stress and anxiety in medical students have been verified in the 1st year of the clerkship [6,44]. More attention should be devoted to the 1st-year clerkship of medical students; in particular, emphasis should be placed on psychological job demands, considering the circumstances of core specialty training (ie, internal medicine, surgery, pediatrics, obstetrics and gynecology, and psychiatry) compared with those involved in the required specialty training (ie, emergency medicine, family medicine, and other subspecialties of internal medicine and surgery), which were indicated to place higher psychological demands in the 1st year of the clerkship than in the 2nd year (*P* < .05).

As expected, both achievement goal motivation orientations of ego and task were beneficial to the clerks’ reduced burnout both in the 1st and 2nd years of the clerkship in this study. Achievement goal motivation orientation scholars have argued that both orientations (ie, task and ego) can be considered orthogonal [45], which can be both high and low in either orientation, or any combinations of low, moderate, or high [46–49]. Moreover, a study reported that task orientation tends to elicit positive psychological responses [50] and that ego orientation is not necessarily a negative motivational quality when moderate to high ego orientation is accompanied by a corresponding level of task orientation [51]. Further examining our sample revealed high average values for task (mean 4.42) and ego (mean 3.93) orientations. This might reasonably explain the positive effects of both achievement orientations on the clerks’ reduced burnout, as observed in our sample.

In this study, we discovered that only the task achievement orientation of the clerks might buffer the negative effects of physical demands on their burnout in the 1st year. Medical students in clerkship devote themselves through participation in the physical, social, and cultural activities of workplaces, and they practice more on procedural tasks as well as experience real-patient encounters and more patient care practice [52]. We might argue that some procedural tasks, including clinical skill simulations, might be new to medical students in clerkship, and novices in clinical workplaces might consider these challenges as requisite learning processes. Therefore, in our study, the clerks’ properties of task achievement goal motivation orientation might drive them to be interested in information relevant to clarifying task demands and promoting mastery [53], thereby buffering the negative effects of physical demands on their burnout to a certain extent. However, regarding the negative effects of training demands (psychological and physical), we observed a limited effect of the task achievement motivation orientation of the medical students only in the 1st year, but not in the 2nd year; therefore, several preventive strategies and preparations must be initiated early, which include clerkship preparation skill courses [54], attitude adjustment [55], and social supports from interpersonal, supervisory, and managerial or organizational stakeholders [56, 57].

Several limitations must be mentioned for this study. One cohort of medical students in one medical school was included in this study. Moreover, with medical students as study samples, the elite and high performance in the academic field in Eastern country contexts might have relatively higher predispositions of achievement goal motivation orientations (the mean for task orientation was 4.42 and that for ego orientation was 3.93 in our study sample), compared with other general student populations at premedical stages based on Taiwanese student entrance examination rankings. The clerks’ burnout levels in this study were regularly measured after their individual clinical specialty rotations. We were able to observe the dynamics of the clerks’ burnout only during their clinical training period, but we were not able to observe the clerks during their preclinical stage. Currently, medical students in clerkships must address both the psychological and physical demands inherent in training workplaces to improve their learning experiences and well-being. In addition to the properties we explored in this study (i.e., achievement goal motivation orientations), future studies involving qualitative or quantitative methods might focus on strategies (based on the identified influencers) that can assist medical students in clerkships to address those demands. The findings must be also validated for professional trainees in other fields, such as pharmacy, nursing, and dentistry, to determine the generalizability of the effect of trainees’ achievement goal motivation on their training.

## Conclusion

Through the ninety-four clerks at a tertiary medical center longitudinally traced with 2230 responses, this study revealed that higher perceived psychological and physical demands of training were related to higher perceived burnout during the 2-year clerkship. Although both the clerks’ task and ego orientations were related to reduced burnout (direct effects), only task orientation was indicated to exert a buffering effect on their perception of physical demands on burnout in the 1st year of the clerkship. Additional studies might focus on strategies to facilitate medical students in clerkships in addressing both the psychological and physical demands inherent in training workplaces to improve their learning experience and well-being.

## Abbreviation

JCQ: Job Content Questionnaire
